# Association of participation in a supplemental nutrition program with stillbirth by race, ethnicity, and maternal characteristics

**DOI:** 10.1186/s12884-018-1920-0

**Published:** 2018-07-24

**Authors:** Meghan Angley, Vanessa R. Thorsten, Carolyn Drews-Botsch, Donald J. Dudley, Robert L. Goldenberg, Robert M. Silver, Barbara J. Stoll, Halit Pinar, Carol J. R. Hogue

**Affiliations:** 1 Department of Epidemiology, Rollins School of Public Health, Emory University, 1518 Clifton Road, Atlanta, GA 30322 USA; 2 Biostatistics and Epidemiology Division, RTI International, Research Triangle Park, North, Carolina USA; 3 Department of Obstetrics and Gynecology, University of Virginia School of Medicine, Charlottesville, Virginia, USA; 4 Department of Obstetrics and Gynecology, Columbia University Medical Center, New York, NY USA; 5 Division of Maternal-Fetal Medicine, Department of Obstetrics and Gynecology, School of Medicine, University of Utah, Salt Lake City, UT USA; 6 McGovern Medical School, University of Texas Health Science Center, Houston, TX USA; 7 The Warren Alpert School of Medicine, Brown University, Providence, RI USA

**Keywords:** Stillbirth, Racial disparities, WIC

## Abstract

**Background:**

Participation in the Special Supplemental Nutrition Program for Women, Infants and Children (WIC) has been associated with lower risk of stillbirth. We hypothesized that such an association would differ by race/ethnicity because of factors associated with WIC participation that confound the association.

**Methods:**

We conducted a secondary analysis of the Stillbirth Collaborative Research Network’s population-based case-control study of stillbirths and live-born controls, enrolled at delivery between March 2006 and September 2008. Weighting accounted for study design and differential consent. Five nested models using multivariable logistic regression examined whether the WIC participation/stillbirth associations were attenuated after sequential adjustment for sociodemographic, health, healthcare, socioeconomic, and behavioral factors. Models also included an interaction term for race/ethnicity x WIC.

**Results:**

In the final model, WIC participation was associated with lower adjusted odds (aOR) of stillbirth among non-Hispanic Black women (aOR: 0.34; 95% CI 0.16, 0.72) but not among non-Hispanic White (aOR: 1.69; 95% CI: 0.89, 3.20) or Hispanic women (aOR: 0.91; 95% CI 0.52, 1.52).

**Conclusions:**

Contrary to our hypotheses, control for potential confounding factors did not explain disparate findings by race/ethnicity. Rather, WIC may be most beneficial to women with the greatest risk factors for stillbirth. WIC-eligible, higher-risk women who do not participate may be missing the potential health associated benefits afforded by WIC.

**Electronic supplementary material:**

The online version of this article (10.1186/s12884-018-1920-0) contains supplementary material, which is available to authorized users.

## Background

The Special Supplemental Nutrition Program for Women, Infants and Children (WIC), established in the 1970’s in the United States, provides assistance in purchasing nutritious foods to pregnant and postpartum women and infants and children up to age five [1]. While there is a long history of research on WIC participation and its association with birth outcomes, study results are mixed, and benefits of WIC may be overstated due to challenges associated with non-random prenatal participation in WIC and gestational age bias [2–4].

Non-random participation of women in WIC is a challenge in assessing the positive impact of WIC on birth outcomes because it is estimated that only two thirds of eligible pregnant or postpartum women participate in WIC [5]. Eligible Black and Hispanic women are more likely to participate than eligible White women. WIC participation is negatively correlated with education, and participation is correlated with non-married status [5]. Further, WIC enrollment criteria stipulate that women be at “nutritional risk,” which includes overweight and underweight women, women with certain conditions, such as diabetes or hypertension, and women with prior adverse pregnancy outcomes [6]. Nearly 70% of pregnant women participating in WIC have more than one nutritional risk factor [1]. Women who choose to participate in WIC may be more health conscious and/or more motivated to prevent adverse outcomes than WIC eligible women who choose not to participate [2]. That said, WIC participation is also associated with smoking during pregnancy among some women [7].

Several recent studies have attempted to address these limitations. Sonchak et al. [8] attempted to control for non-random participation in WIC by examining women who change their WIC status over their reproductive life course. These investigators found WIC to be associated with increased average birth weight and length of gestation and reduced percentage of infants with low birth weight and neonatal intensive care unit admission [8]. Fingar et al. [9], using a fetuses-at-risk approach to address gestational age bias, found that WIC enrollment by gestational week 29 was associated with a lower risk of preterm birth, low birth weight and perinatal death.

Despite conflicting conclusions regarding the overall relationship between WIC and birth outcomes, a consistent finding is that if identified, benefits of WIC are more pronounced among high-risk women. In a study by Sonchak et al., after controlling for gestational age, the association between WIC and lower risk of low birth weight was significant only for Black women [8]. In another study, WIC was protective against fetal death among women with low education and against preterm birth among women with inadequate prenatal care [10]. WIC participation in Kansas was associated with reduced infant mortality among Black and Hispanic, but not White women [11].

While there is some evidence linking WIC to a reduction in fetal death [9, 10], few studies examined racial differences in the association between WIC and risk of stillbirth. In the United States, racial disparities in stillbirth rates persist, with the rate of stillbirths among African American mothers at 10.53 per 1000 live births and stillbirths, compared to 4.88 among White women and 5.22 among Hispanic women in 2013 [12]. Maternal sociodemographic characteristics and nutritional eligibility criteria are associated with stillbirth risk [13], and these factors could also contribute to the observed effect of WIC participation on stillbirth.

The initial objective of our study was to investigate whether the characteristics of women who participate in WIC during pregnancy differ by race or ethnicity. Characteristics examined included comorbid conditions that may affect stillbirth risk, health care utilization during pregnancy, sociodemographic factors and substance use. We then examined if the association between WIC and stillbirth was modified by race or ethnicity and if so, if these differences could be explained by factors associated with participation in WIC. We hypothesized that racial/ethnic differences in the association between WIC and stillbirth would be eliminated by accounting for factors associated with participation in WIC.

## Methods

### Data source

We used data from the Stillbirth Collaborative Research Network (SCRN), a population-based case-control study of stillbirths and live-born controls enrolled at the time of delivery. The design and methods of the study have been previously described [14]. Resident women were enrolled in five selected geographically defined areas (Bristol County, Massachusetts and the state of Rhode Island; DeKalb County, Georgia; Galveston and Brazoria Counties, Texas; Bexar County, Texas and Salt Lake County, Utah) and who delivered between March 2006 and September 2008 at 59 hospitals chosen to capture at least 90% of the resident deliveries. All stillbirths at each site occurring at 18 weeks of gestation or later were selected to be approached for participation in the study. A fetus was considered stillborn if Apgar scores were 0 at 1 and 5 min and there were no signs of life by direct observation. Stillbirths occurring at 18 weeks of delivery up to 20 weeks of delivery were eligible only if they met study criteria and gestational ages were not well dated.

Study personnel screened and registered all livebirths occurring at 20–31 weeks gestation and attempted to enroll controls with pre-defined selection probabilities by week of gestational age in order to enroll adequate numbers of controls born at early gestational ages. For livebirths occurring at greater than or equal to 32 weeks gestational age, random hospital-date-times were selected for each site, and the next eligible delivery in each selected time window was invited to participate. Study personnel gained the permission of women’s physicians before approaching them regarding the study.

Women completed detailed maternal interviews on demographic, behavioral and psychosocial factors. Interviews were largely completed during women’s delivery hospitalizations, but it was possible for women to complete the second half of the interview 2–4 weeks after delivery by phone. When available, medical records were abstracted for information on prenatal care, hospitalizations during pregnancy and the delivery. The study was approved by the institutional review boards of the academic centers, the participating hospitals and the data coordinating center. All participants in the study provided written informed consent.

This secondary analysis was restricted to self-identified non-Hispanic White, non-Hispanic Black and Hispanic women with singleton gestations who completed the maternal interview and for whom medical records were abstracted. Throughout this paper, we refer to these groups as White, Black, and Hispanic.

All demographic information came from the maternal interview. Women were categorized as having chronic hypertension and pre-existing diabetes if the conditions were noted in their chart and/or the women self-reported having these conditions. Pre-pregnancy body mass index (BMI) was determined using the woman’s height and weight recorded in prenatal care medical records. Women with a BMI greater than or equal to 30 kg/m^2^ were considered obese. Trimester of the start of prenatal care was captured from women’s self-reported number of days between becoming pregnant and initiating prenatal care, since information solely from chart abstraction data could misclassify women as starting prenatal care later if chart data from their first prenatal care visit could not be located. Hospitalization prior to the delivery hospitalization was captured by noting any records of hospitalization from chart abstraction.

### Statistical Analyses.

We first examined participation in WIC among stillbirths and live births by maternal characteristics, stratified by race/ethnicity. In addition, to assess the possible impact of gestational age bias, we also examined how gestational age at delivery was related to WIC participation in each race/ethnicity group by using logistic regression to model WIC participation as a function of stillbirth status, controlling for gestational age as a continuous variable. Next, we constructed five nested models to assess the association between WIC participation and stillbirth by race and ethnicity to examine if the relationships were attenuated after adjustment for each set of covariates that have been associated with WIC participation in previous studies. The models were constructed to account sequentially for several categories of factors: 1) factors proximally associated with both stillbirth risk and WIC participation, 2) comorbid conditions, 3) health-care utilization, 4) socio-economic variables and 5) substance use, including smoking and drug use. The five models were as follows:

- Model 1: Adjusted for maternal age, insurance status, gestational age at delivery and pregnancy history.
- Model 2: Adjusted for all variables in Model 1, preexisting diabetes, hypertension and pre-pregnancy obesity.
- Model 3: Adjusted for all variables in Model 2, trimester of entry into prenatal care and chart-documented hospitalizations during pregnancy.
- Model 4: Adjusted for all variables in Model 3, marital status, receipt of wages (by any household member) and education.
- Model 5: Adjusted for all variables in Model 4, smoking status during pregnancy and lifetime illicit drug use.

We first ran the models stratified by race and ethnicity. For each of these models, we noted the total number of observations, by case-control status and by race that we included after dropping observations with missing data. Second, we examined the association between WIC participation and stillbirth with an interaction term for race/ethnicity x WIC to assess differences in the relationship by race/ethnicity. Using these models, we examined both the association between WIC and stillbirth, using the ‘effects’ statement in SAS-Callable SUDAAN to generate race-specific odds ratios and 95% confidence intervals for the association between WIC and stillbirth, as well as the association between race/ethnicity and stillbirth while controlling for all other variables. For all models, we used multivariable logistic regression with generalized estimating equations to account for nine women enrolled twice for two different pregnancies. All models also controlled for catchment area.

We also conducted several sensitivity analyses. First, to address the fact that we did not have data on WIC eligibility in our data, we examined the final model (Model 5) restricted to women who might be eligible for WIC, specifically women who reported having Medicaid or no insurance (Sensitivity A). Next, to examine if the observed associations were robust after restricting to a subset of women with similar prior pregnancy histories, we ran Model 5 restricted to nulliparous women who had experienced no previous pregnancy losses (Sensitivity B). We controlled for gestational age at delivery in Models 1–5, but to remove gestational age as a potential consideration, we restricted Model 5 to deliveries occurring at 37 weeks’ gestation or later without further controlling for gestational age. Furthermore, it has been noted that without controlling for gestational age in a multivariable analysis, it can approximate a fetuses-at-risk approach [15]. Therefore, we also ran Models 1–5 without controlling for gestational age. All statistical analyses were weighted to account for study sampling and differential consent. Analyses were conducted using SAS-Callable SUDAAN software Version 11.0.1 (Research Triangle Institute, Research Triangle Park, NC).

## Results

A weighted sample of 1229 live births and 538 stillbirths was included in this analysis. Among White, Black and Hispanic women with live born infants, 20, 53 and 52%, respectively participated in WIC. Among White, Black and Hispanic women with stillborn infants, 23, 35 and 47%, respectively participated in WIC. We did not have data available for the timing of initiating WIC.

Across all racial/ethnic groups, younger women were more likely to be enrolled in WIC compared with older women (Additional file 1). Among women with chronic hypertension, diabetes or obesity, White women were less likely to participate in WIC than Black and Hispanic women, though these conditions themselves were not associated with WIC participation within racial/ethnic groups. Black and Hispanic women who were married, received household wages or had 13 or more years of education were more likely to be enrolled in WIC than their White counterparts.

Gestational age was most prominently associated with WIC participation among White women. Among live births to White women, a greater proportion of women with deliveries at 32 weeks or later participated in WIC, compared to women with deliveries earlier than 32 weeks. Also among White women, stillborn status was associated with WIC enrollment (OR: 2.23; 95% CI: 1.23, 3.96) while controlling for gestational age, but this was not true among Black or Hispanic women (Table 1).

**Table 1:** Proportion of women reporting enrollment in WIC by race/ethnicity, gestational age and stillbirth status

	White Live Born (*N* = 641)^b^		White Stillborn (*N* = 205)^b^		Black Live Born (*N* = 331)^b^		Black Stillborn (*N* = 123)^b^		Hispanic Live Born (*N* = 666)^b^		Hispanic Stillborn (*N* = 205)^b^	
Gestational Age	Weighted N	% WIC	Weighted N	% WIC	Weighted N	% WIC	Weighted N	% WIC	Weighted N	% WIC	Weighted N	% WIC
< 32 weeks	7	15.2	113	16.8	5	49.7	102	34.9	10	44.9	123	44.3
32+ weeks	589	19.6	80	32.7	147	52.7	32	34.0	471	52.1	88	50.0
Odds Ratio^a^ (95% CI)	2.21 (1.23, 3.96)				0.58 (0.29, 1.16)				0.94 (0.59, 1.50)			

^a^Odds ratio for WIC enrollment, as a function of stillborn/live born status controlling for gestational age in weeks (continuous)

^b^Unweighted counts

In the unadjusted model stratified by race, the strongest association between WIC and stillbirth was among Black women, with reduced odds of stillbirth (OR: 0.49; 95% CI: 0.31, 0.77). Across the five nested models stratified by race, WIC participation was associated with reduced odds of stillbirth among Black women and was not associated with stillbirth among Hispanic women (Table 2). Adjustment across all models sequentially reduced the elevated risk of stillbirth for White women enrolled in WIC, but had little effect on the odds ratios for either Black or Hispanic women (Table 2). When all observations are included in the same models with an interaction term for maternal race/ethnicity x WIC on the odds of stillbirth, similar trends are apparent (Table 3). There appears to be no association between WIC participation and stillbirth among Hispanic women, while WIC participation was associated with reduced odds of stillbirth among Black women across all five models, and greater odds of stillbirth among White women, though the association was attenuated and became imprecise as variables were added to the models. In the final model (Model 5), WIC participation was significantly associated with lower odds of stillbirth among Black women (aOR: 0.34; 95% CI 0.16, 0.72) but not White (aOR: 1.69; 95% CI: 0.89, 3.20) or Hispanic women (aOR: 0.91; 95% CI 0.52, 1.52) (Table 3).

**Table 2:** Association between WIC participation and stillbirth, stratified by race

	White non-Hispanic				Black non-Hispanic				Hispanic			
Model	Live births^a^	Stillbirths^a^	OR	95% CI	Live births^a^	Stillbirths^a^	OR	95% CI	Live births^a^	Stillbirths^a^	OR	95% CI
Unadjusted^b^	593	191	1.33	0.87, 2.02	151	134	0.49	0.31, 0.77	480	211	0.84	0.60, 1.16
Model 1^c^	593	191	2.21	1.06, 4.59	151	130	0.32	0.15, 0.69	479	211	1.32	0.80, 2.18
Model 2	592	189	2.13	1.03, 4.43	147	130	0.33	0.15, 0.71	468	206	1.13	0.67, 1.89
Model 3	588	188	2.15	1.03, 4.48	147	128	0.35	0.16, 0.78	466	204	1.17	0.69, 1.99
Model 4	585	188	1.66	0.74, 3.73	147	126	0.33	0.15, 0.74	463	204	1.16	0.68, 1.96
Model 5	580	185	1.49	0.66, 3.35	147	123	0.31	0.14, 0.68	458	204	1.14	0.67, 1.94
Sensitivity A^d^	161	52	2.11	0.75, 5.95	102	77	0.23	0.09, 0.59	286	133	2.16	1.06, 4.37
Sensitivity B	199	65	0.22	0.06, 0.85	47	36	0.80	0.14, 4.47	115	79	0.99	0.33, 2.92

^a^Weighted counts

^b^Unadjusted associations control only for catchment area

^c^ Model 1: Adjusted for maternal age, insurance status, gestational age at delivery and pregnancy history

Model 2: Adjusted for all variables in Model 1, preexisting diabetes, hypertension and pre-pregnancy obesity

Model 3: Adjusted for all variables in Model 2, trimester of entry into prenatal care and chart-documented hospitalizations during pregnancy

Model 4: Adjusted for all variables in Model 3, marital status, receipt of wages (by any household member) and education

Model 5: Adjusted for all variables in Model 4, smoking status during pregnancy and lifetime illicit drug use

^d^ A: Model 5 restricted to women on Medicaid or without insurance

B: Model 5 restricted to nulliparous women who had experienced no previous pregnancy losses

**Table 3:** Association between WIC and stillbirth by maternal race in models with interaction terms for WIC x maternal race

	White non-Hispanic		Black non-Hispanic		Hispanic	
Model^a^	Odds Ratio	95% CI	Odds Ratio	95% CI	Odds Ratio	95% CI
Model 1	2.23	1.22, 4.10	0.32	0.15, 0.67	1.10	0.66, 1.85
Model 2	2.11	1.14, 3.89	0.32	0.15, 0.66	0.92	0.55, 1.53
Model 3	2.14	1.15, 3.99	0.32	0.15, 0.68	0.93	0.56, 1.55
Model 4	1.92	1.01, 3.64	0.32	0.15, 0.68	0.88	0.53, 1.48
Model 5	1.69	0.89, 3.20	0.34	0.16, 0.72	0.91	0.54, 1.52
Sensitivity A^b^	2.20	0.87, 5.61	0.21	0.08, 0.54	1.62	0.86, 3.04
Sensitivity B	1.00	0.33, 3.02	0.86	0.22, 3.33	0.96	0.34, 2.67

^a^ Model 1: Adjusted for maternal age, insurance status, gestational age at delivery and pregnancy history

Model 2: Adjusted for all variables in Model 1, preexisting diabetes, hypertension and pre-pregnancy obesity

Model 3: Adjusted for all variables in Model 2, trimester of entry into prenatal care and chart-documented hospitalizations during pregnancy

Model 4: Adjusted for all variables in Model 3, marital status, receipt of wages (by any household member) and education

Model 5: Adjusted for all variables in Model 4, smoking status during pregnancy and lifetime illicit drug use

^b^ A: Model 5 restricted to women on Medicaid or without insurance

B: Model 5 restricted to nulliparous women who had experienced no previous pregnancy losses

Using these same models, associations between race and stillbirth for both women not on WIC and women participating in WIC are depicted in Table 4, again using the ‘effects’ statement to generate odds ratios and 95% confidence intervals. Among women not receiving WIC, there were consistently higher odds of stillbirth for Black and for Hispanic women in Models 1–3 compared to White women. Among women receiving WIC during pregnancy, the odds ratios for the association of maternal race and stillbirth were imprecise, but were not significantly elevated among Black and Hispanic women compared to White women.

**Table 4:** Association between maternal race and stillbirth in models with interaction terms for WIC x maternal race, stratified by WIC status

	WIC = 0 (not enrolled)		WIC = 1 (enrolled)	
Model	Odds Ratio	95% CI	Odds Ratio	95% CI
Model 1^a^				
Black vs. White	4.12	2.11, 8.08	0.59	0.27, 1.29
Hispanic vs. White	1.67	1.02, 2.73	0.82	0.44, 1.56
Model 2				
Black vs. White	3.84	1.98, 7.42	0.58	0.26, 1.27
Hispanic vs. White	1.85	1.14, 3.02	0.81	0.43, 1.55
Model 3				
Black vs. White	3.82	1.97, 7.41	0.57	0.26, 1.28
Hispanic vs. White	1.80	1.10, 2.95	0.78	0.41, 1.50
Model 4				
Black vs. White	3.51	1.78, 6.92	0.59	0.27, 1.31
Hispanic vs. White	1.59	0.94, 2.66	0.73	0.37, 1.43
Model 5				
Black vs. White	3.38	1.70, 6.71	0.67	0.30, 1.51
Hispanic vs. White	1.68	0.99, 2.85	0.90	0.46, 1.77
Sensitivity A^b^				
Black vs. White	4.78	1.67, 13.69	0.46	0.17, 1.27
Hispanic vs. White	1.12	0.44, 2.82	0.82	0.38, 1.80
Sensitivity B				
Black vs. White	1.43	0.42, 4.91	1.23	0.34, 4.50
Hispanic vs. White	2.20	0.90, 5.39	2.11	0.57, 7.74

^a^ Model 1: Adjusted for maternal age, insurance status, gestational age at delivery and pregnancy history

Model 2: Adjusted for all variables in Model 1, preexisting diabetes, hypertension and pre-pregnancy obesity

Model 3: Adjusted for all variables in Model 2, trimester of entry into prenatal care and chart-documented hospitalizations during pregnancy

Model 4: Adjusted for all variables in Model 3, marital status, receipt of wages (by any household member) and education

Model 5: Adjusted for all variables in Model 4, smoking status during pregnancy and lifetime illicit drug use

^b^ A: Model 5 restricted to women on Medicaid or without insurance

B: Model 5 restricted to nulliparous women who had experienced no previous pregnancy losses

Restricting Model 5 in Table 3 to term deliveries did not change the direction and magnitude of the relationships. Not controlling for gestational age in Models 1–5 did not change the fact that WIC appeared protective among Black women in all Models, but among White women, the associations were pulled to the null. For example, an odds ratio of 1.48 (95% CI: 0.95, 2.30) compared to an odds ratio of 2.23 (95% CI: 1.22, 4.10) in Table 3, Model 1. In addition, restricting Model 5 to women who are most likely to be eligible for WIC (Sensitivity A) showed similar trends, with WIC being associated with reduced odds of stillbirth among Black women and no association among White or Hispanic women (Tables 2-3). However, when Model 5 was restricted to nulliparous women with no previous losses, the racial/ethnic differences in the association between WIC and stillbirth were eliminated, with the respective adjusted odds ratios for White, Black and Hispanic women as 1.00 (95% CI 0.33, 3.02), 0.86 (95% CI 0.22, 3.33) and 0.96 (95% CI 0.34, 2.67) (Table 3).

## Discussion

Our findings are consistent with studies of WIC participation and outcomes other than stillbirth, in which positive outcomes appear more pronounced among Black women enrolled in WIC compared to other racial/ethnic groups [8, 11]. This racial/ethnic difference in outcome by WIC status held true after controlling for factors that may be associated with participation in WIC. These relationships also remained consistent when restricting the sample to deliveries occurring at 37 weeks’ gestation or later, restricting to women who had Medicaid or no insurance and not controlling for gestational age at delivery. However, when restricting to nulliparous women with no previous losses, racial/ethnic differences in the association between WIC and stillbirth were eliminated.

Although we controlled for a number of factors that may be associated with participation in WIC and confounders of the relationship between WIC and stillbirth, the observed relationship may be due to unmeasured confounders. Residual confounding may also differ between racial/ethnic groups. White women who enroll in WIC may have a disproportionate distribution of unmeasured risk factors for stillbirth. In a study of WIC-eligible women, higher income was associated with a decrease in WIC participation and unintended pregnancy was associated with increased participation in WIC only among White women [16]. This was also true in our population, where among live births to women who received household wages, only 17.2% of White women participated in WIC compared to 50% of their Black and Hispanic counterparts. Generally, families that are eligible but choose not to enroll in WIC have more economic and personal resources [17], but one possibility is that among White women these economic and personal resources do not translate to a reduced risk of stillbirth.

Khanani et al. also found that while the infant mortality rate was reduced among Black WIC participants compared to non-participants, there was very little association among White WIC participants [7]. The authors suggest that this may be due to the high rate of smoking among White WIC participants. WIC enrollment was also associated with a slightly higher smoking relapse rate among White Women compared to Black women [18]. While we controlled for both smoking during pregnancy and lifetime illicit drug use, which in our data were associated with WIC enrollment among White but not Black or Hispanic women, this did not change the observation that WIC appeared protective among Black women and was associated with stillbirth among White women. This positive association may be due to unmeasured confounding among the White women. A much lower proportion of White women compared to Black women participated in WIC (20% vs. 53% among live born controls), and it is likely that White women who participated in WIC were systematically different from White women who did not, with those who participated being at greater risk of stillbirth. Additionally, the positive association between WIC and stillbirth is substantially reduced when gestational age is not included in the models. In other words, the positive association between WIC and stillbirth among White women exists only within categories of gestational age. It has been suggested that racial/ethnic differences in induction of labor may be partly responsible for the racial disparity in stillbirth [19]. Within categories of gestational age, White women at risk of stillbirth and who are not on WIC may be more likely to be induced or have aggressively managed deliveries due to a combination of clinical indicators and demographic factors, compared to White women participating in WIC.

The protective association between WIC and stillbirth among Black women may also be due to residual confounding, where Black women who choose to participate in WIC are more health-conscious or proactive in addressing stillbirth risk factors than Black women who do not participate in WIC. Alternatively, WIC may be most beneficial to women with the specific risk factors for stillbirth, and Black women may represent this group due to a combination of preexisting conditions and access to medical care. Many factors have been shown to contribute, at least in small part, to the racial disparity in stillbirth [20]. Black and Hispanic women are more likely than White women to enter pregnancy with chronic conditions that increase the risk of stillbirth, including diabetes, hypertension, obesity and autoimmune disorders [21]. Overall, maternal health conditions contribute more to the hazard of stillbirth among Black women than among White and Hispanic women [22]. In our study, greater proportions of Black women who were obese, had diabetes or chronic hypertension enrolled in WIC compared to their White counterparts. For these Black women at a particularly high risk of stillbirth, the nutritional and social support that WIC provided may have contributed to averting a stillbirth, and one could speculate that we might have seen a similar association among White women had more high-risk White women participated in WIC prenatally. While the exact pathways through which WIC is associated with positive outcomes are unknown, evidence suggests it may be through favorable gestational weight gain patterns and better connections to medical services, both of which may serve to improve outcomes among women with chronic conditions [23].

The sensitivity analyses demonstrated that Medicaid status did not account for the supposed differential sorting of Black and White women into WIC, nor did restricting the study sample to women who had delivered at 37 weeks’ gestation or later. However, racial/ethnic differences in the association between WIC participation and stillbirth were eliminated when the analysis was restricted to nulliparous women with no previous losses, and all associations were pulled toward the null. Any nulliparity and multiparity with previous losses are associated with greater odds of stillbirth compared to women who have already experienced pregnancies without losses [13]. Further restricting to nulliparous women with no losses also excludes women who enrolled in WIC because they met the criteria for nutritional risk because they experienced previous losses [1]. Therefore, restricting to women who are all in their first pregnancy may represent a particularly homogenous group with fewer unmeasured confounders.

Our study had several important limitations. First, we did not have information on which participants in our study were actually eligible for WIC. However, we attempted to address this issue in our sensitivity analysis, which mirrored our original results. Several other studies have also addressed this issue by restricting their study sample to women receiving Medicaid [8, 9]. Second, we lacked information on timing of WIC enrollment and redemption of vouchers. Over half of pregnant women enrolling in WIC do so in the first trimester [1], and there was no evidence to suggest that timing of enrollment differs by race/ethnicity. We also attempted to reduce gestational age bias, where pregnancies with a longer gestation are less likely to result in stillbirth and have more time to enroll in WIC prenatally, by controlling for gestational age at delivery, though this can be problematic [24]. We were unable to use a true fetuses-at-risk approach as we did not have information on timing of WIC enrollment and could not construct exposed and unexposed cohorts, though we did attempt to approximate this analysis by not controlling for gestational age at delivery. A study that used a fetuses-at-risk approach showed that WIC participation was associated with favorable birth outcomes depending on the gestational week of enrollment [9]. We were also limited in our ability to precisely estimate some associations. In the sensitivity analyses which further restricted sample sizes, the confidence intervals were particularly wide. Finally, the question we used to determine WIC participation referred to the woman’s sources of household income in the prior 12 months, so there was the possibility that women who responded affirmatively were not enrolled prenatally during the index pregnancy, but were rather enrolled because they were breastfeeding or in the postpartum period from a prior pregnancy. However, we did not note any significant differences in WIC participation by pregnancy history for any race/ethnicity. Important strengths of this study included the use of a population-based dataset that was designed to examine stillbirth, a relatively rare birth outcome. As such, our study is one of only a handful of studies that specifically examine the association between WIC and stillbirth. We also were able to include selected clinical data in our analyses.

## Conclusions

Our study suggests that WIC participation is associated with decreased risk of stillbirth among Black women. We believe WIC enrollment would be helpful for all WIC-eligible women, and that the null associations found for White and Hispanic women in this study are likely due to additional unmeasured factors associated with participation among these women.

## Additional file

Additional file 1: “Weighted characteristics by race, and percent of each subgroup enrolled in WIC” provides the proportion of women with certain demographic characteristics participating in WIC. Proportions are presented separately by race/ethnicity. (DOCX 25 kb)

## Abbreviations

BMI: Body mass index
OR: Odds ratio
WIC: Special Supplemental Nutrition Program for Women, Infants and Children
