# Supplementary material for: Association of participation in a supplemental nutrition program with stillbirth by race, ethnicity, and maternal characteristics
Source: BMC Pregnancy Childbirth. 2018 Jul 24;18:306. doi: 10.1186/s12884-018-1920-0 (PMC6056947; doi:10.1186/s12884-018-1920-0)
Supplement: Supplementary file 1 — “Weighted characteristics by race, and percent of each subgroup enrolled in WIC” provides the proportion of women with certain demographic characteristics participating in WIC. Proportions are presented separately by race/ethnicity. (DOCX 25 kb) [file 12884_2018_1920_MOESM1_ESM.docx]

Additional file 1

Table S1. Weighted characteristics by race, and percent of each subgroup enrolled in WIC

|  | White Live Births | | White Stillbirths | | Black Live Births | | Black Stillbirths | | Hispanic Live Births | | Hispanic Stillbirths | |
| --- | --- | --- | --- | --- | --- | --- | --- | --- | --- | --- | --- | --- |
|  | Weighted N | % in WIC | Weighted N | % in WIC | Weighted N | % in WIC | Weighted N | % in WIC | Weighted N | % in WIC | Weighted N | % in WIC |
| **Maternal age** |  |  |  |  |  |  |  |  |  |  |  |  |
| <20 | 31 | 62.6 | 15 | 57.8 | 25 | 72.9 | 19 | 41.2 | 79 | 71.1 | 40 | 61.8 |
| 20-34 | 453 | 20.0 | 142 | 22.4 | 112 | 49.8 | 96 | 33.1 | 356 | 49.9 | 138 | 45.7 |
| 35-39 | 93 | 6.2 | 27 | 10.9 | 11 | 35.5 | 14 | 41.0 | 37 | 31.5 | 25 | 35.2 |
| 40+ | 16 | 0.0 | 7 | 17.9 | 3 | 45.7 | 5 | 19.5 | 8 | 47.8 | 9 | 27.1 |
| **Gestational Age** |  |  |  |  |  |  |  |  |  |  |  |  |
| 18-23 | 1 | 20.8 | 65 | 13.7 | 1 | 52.0 | 70 | 23.8 | 2 | 39.2 | 62 | 49.3 |
| 24-27 | 2 | 20.1 | 29 | 17.2 | 2 | 49.3 | 13 | 75.2 | 4 | 45.9 | 34 | 32.5 |
| 28-31 | 4 | 11.8 | 18 | 27.3 | 2 | 48.3 | 19 | 48.9 | 5 | 45.9 | 27 | 47.5 |
| 32-36 | 36 | 26.7 | 42 | 28.5 | 18 | 66.7 | 15 | 41.7 | 51 | 54.9 | 49 | 45.0 |
| 37+ | 550 | 19.1 | 36 | 37.6 | 129 | 50.7 | 17 | 26.9 | 419 | 51.8 | 39 | 56.4 |
| **Pregnancy History** |  |  |  |  |  |  |  |  |  |  |  |  |
| Nulliparous (no losses) | 204 | 23.3 | 65 | 19.9 | 49 | 56.0 | 40 | 34.6 | 119 | 43.8 | 79 | 36.8 |
| Nulliparous (previous losses) | 36 | 8.7 | 23 | 17.4 | 7 | 61.8 | 16 | 36.8 | 21 | 60.4 | 16 | 40.8 |
| Multiparous (no losses) | 247 | 20.9 | 64 | 27.1 | 70 | 48.7 | 50 | 32.3 | 244 | 55.9 | 61 | 55.4 |
| Multiparous (losses, no stillbirth) | 100 | 12.6 | 27 | 26.7 | 20 | 53.6 | 16 | 36.8 | 91 | 49.7 | 41 | 46.1 |
| Multiparous (previous stillbirth) | 7 | 12.3 | 12 | 25.9 | 6 | 56.0 | 12 | 38.8 | 6 | 60.5 | 15 | 70.9 |
| **Insurance** |  |  |  |  |  |  |  |  |  |  |  |  |
| Medicaid/None | 165 | 53.9 | 54 | 57.7 | 106 | 60.9 | 81 | 40.5 | 301 | 62.7 | 137 | 57.9 |
| Other | 428 | 6.2 | 137 | 9.8 | 46 | 33.4 | 50 | 25.0 | 178 | 34.2 | 73 | 25.1 |
| **Diabetes** |  |  |  |  |  |  |  |  |  |  |  |  |
| Yes | 9 | 10.7 | 9 | 31.8 | 2 | 0.0 | 9 | 22.9 | 8 | 46.3 | 11 | 72.4 |
| No | 583 | 19.7 | 182 | 22.9 | 149 | 52.8 | 125 | 35.5 | 472 | 52.1 | 200 | 45.2 |
| **Hypertension** |  |  |  |  |  |  |  |  |  |  |  |  |
| Yes | 35 | 18.7 | 19 | 14.7 | 12 | 40.5 | 18 | 46.8 | 27 | 55.5 | 15 | 56 |
| No | 558 | 19.6 | 172 | 24.3 | 140 | 53.6 | 116 | 32.8 | 453 | 51.8 | 197 | 46 |
| **Obese** |  |  |  |  |  |  |  |  |  |  |  |  |
| Yes | 113 | 25.8 | 57 | 28.4 | 34 | 43.6 | 41 | 40.6 | 137 | 51.7 | 70 | 40.5 |
| No | 479 | 17.8 | 132 | 21.4 | 114 | 55.9 | 93 | 31.5 | 332 | 53.0 | 137 | 48.7 |
| **Prenatal Care First Trimester** |  |  |  |  |  |  |  |  |  |  |  |  |
| Yes | 538 | 18.7 | 168 | 20.9 | 115 | 51.1 | 83 | 35.9 | 381 | 51.4 | 154 | 46.3 |
| No | 51 | 27.7 | 21 | 39.7 | 36 | 57.1 | 49 | 33.9 | 97 | 54.8 | 55 | 46.1 |
| **Hospitalization** |  |  |  |  |  |  |  |  |  |  |  |  |
| Yes | 36 | 36.1 | 12 | 25.9 | 11 | 44.3 | 14 | 37.9 | 25 | 57.3 | 14 | 72.5 |
| No | 556 | 18.4 | 179 | 23.2 | 140 | 53.2 | 120 | 34.3 | 455 | 51.7 | 198 | 44.9 |
| **Marital Status** |  |  |  |  |  |  |  |  |  |  |  |  |
| Neither | 57 | 57.7 | 23 | 54.6 | 55 | 57.7 | 51 | 40.5 | 79 | 65.8 | 58 | 50.5 |
| Cohabitating | 97 | 49.3 | 38 | 42.4 | 47 | 57.4 | 41 | 33.9 | 152 | 56.6 | 63 | 55.1 |
| Married | 439 | 8.0 | 130 | 12.3 | 49 | 42.1 | 42 | 28.3 | 249 | 44.8 | 90 | 38.4 |
| **Education** |  |  |  |  |  |  |  |  |  |  |  |  |
| 0-11 | 36 | 57.6 | 22 | 48.6 | 26 | 65.8 | 26 | 47.0 | 175 | 57.7 | 84 | 61.0 |
| 12 | 112 | 43.2 | 46 | 41.7 | 58 | 57.8 | 44 | 33.7 | 153 | 58.7 | 70 | 43.7 |
| 13+ | 443 | 10.3 | 122 | 11.8 | 68 | 43.0 | 62 | 31.4 | 149 | 38.0 | 58 | 29.6 |
| **Wages** |  |  |  |  |  |  |  |  |  |  |  |  |
| Yes | 561 | 17.2 | 181 | 20.3 | 132 | 50.0 | 111 | 32.1 | 433 | 50.3 | 181 | 45.1 |
| No | 32 | 61.4 | 10 | 78.9 | 19 | 69.9 | 23 | 46.9 | 47 | 67.8 | 30 | 56.6 |
| **Smoked during pregnancy** |  |  |  |  |  |  |  |  |  |  |  |  |
| Yes | 66 | 46.1 | 44 | 48.5 | 20 | 46.2 | 19 | 21.5 | 18 | 67.4 | 15 | 63.9 |
| No | 527 | 16.2 | 146 | 15.7 | 131 | 53.5 | 115 | 36.3 | 461 | 51.3 | 196 | 45.3 |
| **Lifetime illicit drug use** |  |  |  |  |  |  |  |  |  |  |  |  |
| Yes | 219 | 28.0 | 86 | 27.6 | 47 | 45.5 | 46 | 37.8 | 91 | 51.6 | 40 | 40.8 |
| No | 368 | 14.8 | 102 | 19.2 | 103 | 55.2 | 87 | 33.7 | 377 | 52.9 | 170 | 47.8 |
